# EAACI guidelines on the management of IgE‐mediated food allergy

**DOI:** 10.1111/all.16345

**Published:** 2024-10-30

**Authors:** Alexandra F. Santos, Carmen Riggioni, Ioana Agache, Cezmi A. Akdis, Mubeccel Akdis, Alberto Alvarez‐Perea, Montserrat Alvaro‐Lozano, Barbara Ballmer‐Weber, Simona Barni, Kirsten Beyer, Carsten Bindslev‐Jensen, Helen A. Brough, Betul Buyuktiryaki, Derek Chu, Stefano Del Giacco, Audrey Dunn‐Galvin, Bernadette Eberlein, Motohiro Ebisawa, Philippe Eigenmann, Thomas Eiwegger, Mary Feeney, Montserrat Fernandez‐Rivas, Alessandro Fiocchi, Helen R. Fisher, David M. Fleischer, Mattia Giovannini, Claudia Gray, Karin Hoffmann‐Sommergruber, Susanne Halken, Jonathan O'B Hourihane, Christina J. Jones, Marek Jutel, Edward F. Knol, George N. Konstantinou, Gideon Lack, Susanne Lau, Andreina Marques Mejias, Mary Jane Marchisotto, Rosan Meyer, Charlotte G. Mortz, Beatriz Moya, Antonella Muraro, Caroline Nilsson, Lucila Camargo Lopes de Oliveira, Liam O'Mahony, Nikolaos G. Papadopoulos, Kirsten P. Perrett, Rachel Peters, Marcia Podesta, Lars K. Poulsen, Graham Roberts, Hugh Sampson, Jürgen Schwarze, Peter Smith, Elizabeth Tham, Eva Untersmayr, Ronald Van Ree, Carina Venter, Brian Vickery, Berber Vlieg‐Boerstra, Thomas Werfel, Margitta Worm, George Du Toit, Isabel Skypala

**Affiliations:** ^1^ Department of Women and Children's Health (Pediatric Allergy), School of Life Course Sciences, Faculty of Life Sciences and Medicine King's College London London UK; ^2^ Peter Gorer Department of Immunobiology, School of Immunology and Microbial Sciences King's College London London UK; ^3^ Children's Allergy Service, Evelina London Children's Hospital Guy's and St Thomas' Hospital London UK; ^4^ Division of Immunology and Allergy The Hospital for Sick Children and the SickKids Food Allergy and Anaphylaxis Program Toronto Ontario Canada; ^5^ Department of Paediatrics, Temerty Faculty of Medicine University of Toronto Toronto Ontario Canada; ^6^ Faculty of Medicine Transylvania University Brasov Romania; ^7^ Swiss Institute of Allergy and Asthma Research (SIAF) University Zurich Davos Switzerland; ^8^ Hospital General Universitario Gregorio Marañón Madrid Spain; ^9^ Gregorio Marañón Health Research Institute Madrid Spain; ^10^ Pediatric Allergy and Clinical Immunology Department Hospital Sant Joan de Déu Barcelona Spain; ^11^ Institut de Recerca Sant Joan de Déu Universitat de Barcelona Barcelona Spain; ^12^ Clinic for Dermatology and Allergology Kantonsspital St. Gallen St. Gallen Switzerland; ^13^ Department of Dermatology University Hospital Zurich Zurich Switzerland; ^14^ Allergy Unit Meyer Children's Hospital IRCCS Florence Italy; ^15^ Department of Pediatric Respiratory Medicine, Immunology and Critical Care Medicine Charité Universitätsmedizin Berlin Berlin Germany; ^16^ Department of Dermatology and Allergy Centre, Odense Research Centre for Anaphylaxis (ORCA), Odense University Hospital University of Southern Denmark Odense Denmark; ^17^ Department of Pediatrics, Division of Pediatric Allergy Koc University School of Medicine Istanbul Türkiye; ^18^ McMaster University Hamilton Canada; ^19^ Department of Medical Sciences and Public Health and Unit of Allergy and Clinical Immunology, University Hospital “Duilio Casula” University of Cagliari Cagliari Italy; ^20^ Paediatrics and Child Health, INFANT Centre, HRB‐CRF University College Cork Cork Ireland; ^21^ Paediatrics and Child Health, Royal College of Surgeons in Ireland Children's Health Ireland Dublin Ireland; ^22^ Department of Dermatology and Allergy Technical University of Munich, School of Medicine Munich Germany; ^23^ Clinical Research Center for Allergy and Rheumatology NHO Sagamihara National Hospital Kanagawa Japan; ^24^ Department of Pediatrics, Gynecology and Obstetrics University Hospitals of Geneva Geneva Switzerland; ^25^ Karl Landsteiner University of Health Sciences Krems an der Donau Austria; ^26^ Department of Pediatric and Adolescent Medicine University Hospital St. Pölten St.Pölten Austria; ^27^ Translational Medicine Program, Research Institute Hospital for Sick Children Toronto Ontario Canada; ^28^ Department of Immunology, Temerty Faculty of Medicine University of Toronto Toronto Ontario Canada; ^29^ Allergy Department Hospital Clinico San Carlos Madrid Spain; ^30^ Facultad de Medicina Universidad Complutense, IdISSC, ARADyAL Madrid Spain; ^31^ Allergy Department Pediatric Hospital Bambino Gesù IRCCS Rome Italy; ^32^ University of Colorado School of Medicine and Children's Hospital Colorado Aurora Colorado USA; ^33^ Department of Health Sciences University of Florence Florence Italy; ^34^ Red Cross Children's Hospital and Kidsallergy Centre Cape Town South Africa; ^35^ Department of Paediatrics University of Cape Town Cape Town South Africa; ^36^ Dept. of pathophysiology and Allergy Research Medical University of Vienna Vienna Austria; ^37^ Hans Christian Andersen Children's Hospital Odense University Hospital Odense Denmark; ^38^ Royal College of Surgeons in Ireland and Childrens Health Ireland Dublin Ireland; ^39^ School of Psychology, Faculty of Health and Medical Sciences University of Surrey Guildford UK; ^40^ Department of Clinical Immunology, Faculty of Medicine Wrocław Medical University; and ALL‐MED Medical Research Institute Wroclaw Poland; ^41^ Department Center of Translational Immunology and Department Dermatology/Allergology University Medical Center Utrecht Utrecht The Netherlands; ^42^ Department of Allergy and Clinical Immunology 424 General Military Training Hospital Thessaloniki Greece; ^43^ EAACI Patient Organisation Committee Zurich Switzerland; ^44^ MJM Advisory New York New York USA; ^45^ Dept. Nutrition and Dietetics Winchester University Winchester UK; ^46^ Department of Medicine KU Leuven Leuven Belgium; ^47^ Department of Allergy Hospital Universitario 12 de Octubre Madrid Spain; ^48^ Instituto de Investigación Sanitaria Hospital 12 de Octubre (imas12) Madrid Spain; ^49^ Food Allergy Referral Centre Padua University Hospital Padua Italy; ^50^ Department of Clinical Science and Education Karolinska Institutet Solna Sweden; ^51^ Sachs Children and Youth Hospital South Hospital Stockholm Sweden; ^52^ Division of Allergy and Clinical Immunology, Department of Paediatrics Federal University of São Paulo São Paulo Brazil; ^53^ Department of Medicine, School of Microbiology, APC Microbiome Ireland University College Cork Cork Ireland; ^54^ Allergy Dpt, 2nd Pediatric Clinic University of Athens Athens Greece; ^55^ Lydia Becker Institute University of Manchester Manchester UK; ^56^ Population Allergy Murdoch Children's Research Institute Parkville Australia; ^57^ Department of Paediatrics University of Melbourne Parkville Victoria Australia; ^58^ Department of Allergy and Immunology Royal Children's Hospital Parkville Australia; ^59^ Murdoch Children's Research Institute Parkville Victoria Australia; ^60^ Department of Paediatrics the University of Melbourne Parkville Victoria Australia; ^61^ European Federation of Allergy and Airways Diseases Patients' Associations and the EAACI Patient Organisation Committee Zurich Switzerland; ^62^ Allergy Clinic Copenhagen University Hospital at Herlev‐Gentofte Copenhagen Denmark; ^63^ Paediatric Allergy and Respiratory Medicine, University of Southampton, NIHR Southampton Biomedical Research Centre and David Hide Asthma and Allergy Centre St Mary Hospital Isle of Wight UK; ^64^ Department of Pediatrics, Division of Allergy and Immunology, Jaffe Food Allergy Institute Icahn School of Medicine at Mount Sinai New York New York USA; ^65^ Child Life and Health, Centre for Inflammation Research, Institute for Regeneration and Repair The University of Edinburgh Edinburgh UK; ^66^ Clinical Medicine Griffith University Southport Queensland Australia; ^67^ Queensland Allergy Services Private Practice Southport Queensland Australia; ^68^ Yong Loo Lin School of Medicine National University of Singapore Singapore Singapore; ^69^ Khoo Teck Puat‐National University Children's Medical Institute National University Health System (NUHS) Singapore Singapore; ^70^ Human Potential Translational Research Programme, Yong Loo Lin School of Medicine National University of Singapore Singapore Singapore; ^71^ Institute of Pathophysiology and Allergy Research, Center of Pathophysiology, Infectiology and Immunology Medical University of Vienna Vienna Austria; ^72^ Department of Experimental Immunology and of Otorhinolaryngology Amsterdam University Medical Centers Amsterdam The Netherlands; ^73^ Section of Allergy and Clinical Immunology, Children's Hospital Colorado University of Colorado Boulder Colorado USA; ^74^ Emory University School of Medicine and Children's Healthcare of Atlanta Atlanta Georgia USA; ^75^ Department of Paediatrics OLVG Hospital Amsterdam the Netherlands; ^76^ Rijnstate Allergy Centre Rijnstate Hospital Arnhem The Netherlands; ^77^ Vlieg Dieticians Private Practice for dietary management of food allergy Arnhem the Netherlands; ^78^ Department of Dermatology and Allergy Hannover Medical School Hannover Germany; ^79^ Part of Guys and St Thomas NHS Foundation Trust Royal Brompton and Harefield Hospitals London UK; ^80^ Department of Inflammation and Repair Imperial College London UK

**Keywords:** allergen immunotherapy, anaphylaxis, biologicals, diet, food allergy, IgE‐mediated food allergy, management, treatment

## Abstract

This European Academy of Allergy and Clinical Immunology (EAACI) guideline provides recommendations for the management of IgE‐mediated food allergy and was developed using the Grading of Recommendations, Assessment, Development and Evaluations (GRADE) approach. Following the confirmation of IgE‐mediated food allergy diagnosis, allergen avoidance and dietary advice (with support of a specialised dietitian, if possible) together with the provision of a written treatment plan, education on the recognition of allergic symptoms and prescription of medication including adrenaline using an auto‐injector are essential. Patients with significant anxiety and requirement for coping strategies may benefit from support from a clinical psychologist. As immunomodulatory interventions, omalizumab is suggested for treatment of IgE‐mediated food allergy in children from the age of 1 and adults; and oral allergen‐specific immunotherapy is recommended for children and adolescents with peanut allergy and suggested for milk and egg allergies (generally after 4 years of age for milk and egg). Sublingual and epicutaneous immunotherapy are suggested for peanut allergy but are not yet available at the point of care. Future research into disease modifying treatments for IgE‐mediated food allergy are highly needed, with standardised and patient‐focused protocols and outcomes.

## 1 INTRODUCTION

**FIGURE 1:**
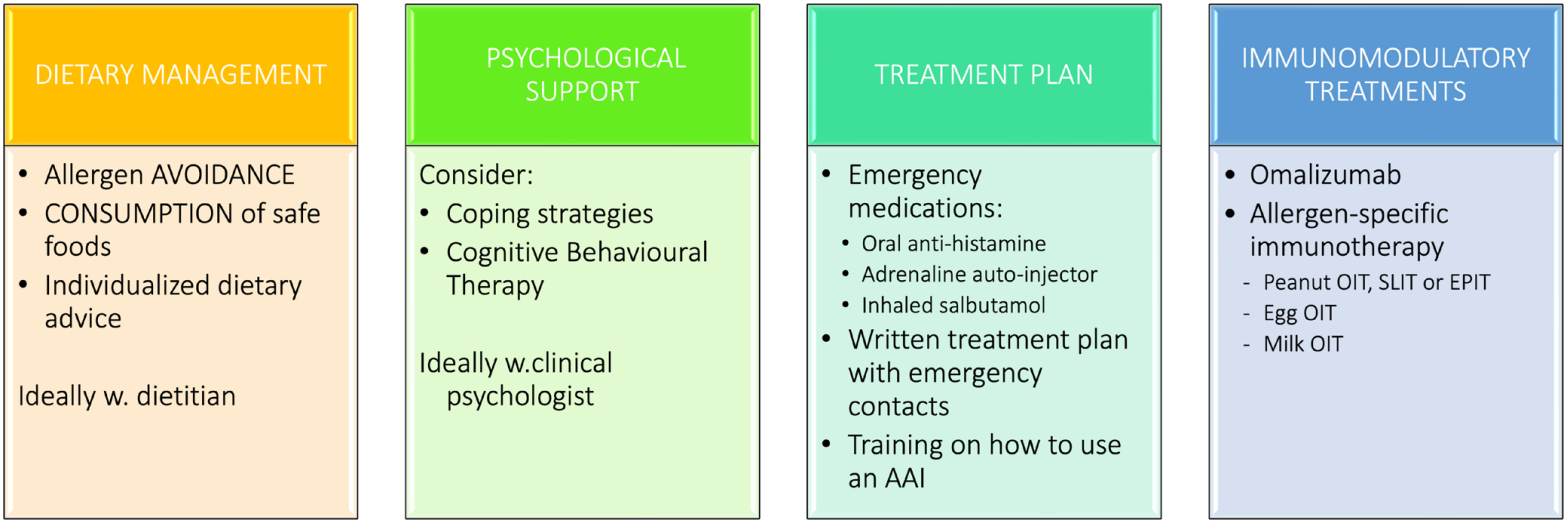
Management of IgE‐mediated food allergy.

IgE‐mediated food allergy affects up to 10% of the population, particularly the younger age groups, who are also at higher risk of nutritional deficiencies as a consequence of unsupervised and unduly prolonged avoidance diets.^1^, ^2^, ^3^ Conversely, young children are also the age group with more opportunities for prevention and disease‐modifying treatments, as their immune response is more plastic and more amenable to immunomodulation. Recently, there has been evolution from passive management of IgE‐mediated food allergy, relying on allergen avoidance, the “watch‐wait approach”, and monitoring for possible spontaneous resolution, towards more active management. This includes the introduction of allergenic foods for prevention, which is covered in separate EAACI guidelines,^4^, ^5^, ^6^ individualised dietary advice (e.g. incorporating baked foods in children with milk/egg/soya allergies) and specific immunomodulatory interventions, such as allergen‐specific immunotherapy and biologicals (Figure 1).

The EAACI Guidelines on IgE‐mediated Food Allergy build on a preceding iteration published in 2014^5^ and on the immunotherapy for IgE‐mediated food allergy guidelines published in 2018.^7^ The updated EAACI Guidelines on IgE‐mediated Food Allergy comprise two parts: the first part, previously published, focused on diagnosis,^6^, ^8^ whilst this second part focuses on clinical management of IgE‐mediated food allergy, including immunomodulatory treatments, that is, allergen‐specific immunotherapy and biologics. This guideline was informed by a dedicated systematic review of the literature and meta‐analyses, already published in the “Allergy” journal.^9^ A critical aspect that became apparent from the systematic review was the heterogeneity of study protocols adopted in the various clinical trials, including primary outcomes, that hampered comparability and standardisation of treatments for clinical implementation—this issue is being covered by a separate EAACI taskforce.^10^ For this guideline, we will use the definitions adopted by the investigators of each individual study, which generally reflect the definitions from the 2018 EAACI guidelines on allergen‐specific immunotherapy for food allergy^7^—see Box 1.

BOX 1 **Definitions used in this EAACI guideline**

***Desensitisation*** is defined as efficacy during treatment, that is, the ability to consume a defined amount of the food without dose‐limiting symptoms while the patient is on the given therapy.

***Remission or sustained unresponsiveness*** is defined as post‐discontinuation efficacy, that is, the ability to consume a defined amount of the food without dose‐limiting symptoms, after stopping treatment for a period of time.

***Tolerance*** is defined as the ability to consume the food without symptoms, regardless of quantity or frequency with which the food is consumed.

## 2 METHODS

### 2.1 Scope of guidelines

The EAACI Food Allergy Guidelines focus on IgE‐mediated food allergy and are aimed at health care professionals specialised in Allergy and Clinical Immunology or a related specialty and primary care practitioners and allied health colleagues who assess and manage patients with food allergy in their daily practice.

### 2.2 Expert group and stakeholder involvement

The EAACI Food Allergy Guidelines were commissioned by EAACI and led by the steering committee chaired by Alexandra F. Santos and formed by Alexandra F. Santos, Isabel Skypala, George Du Toit and Carmen Riggioni. An expert group was formed to advise on the elaboration of the guidelines and formulation of the recommendations, listed as authors. The expert group included authors of the last EAACI Food Allergy Guidelines, current board members of relevant EAACI sections and interest groups, additional experts from countries outside Europe such as USA, Canada, Brazil, South Africa, Hong Kong, Singapore and Australia, to ensure global relevance of the guidelines, and from key areas such as psychology, allied health and EAACI junior members. Patient representatives were included as well, namely from the European Federation of Allergy and Airways Diseases (EFA) and the EAACI Patient Organisations' Committee (POC) to provide input throughout the process from inception to publication and dissemination.

### 2.3 Systematic review of the evidence and formulation of recommendations

The present food allergy guideline was informed by a systematic review of the literature and meta‐analyses^9^ on efficacy and safety of food allergy immunotherapy and biologics. Unless otherwise stated the relative risk (RR) refers to desensitisation. An independent systematic review performed by experts in mental health was used to support recommendations on psychological management.^11^ In the absence of a systematic review, an expert consensus, based on a narrative review of the relevant evidence‐based approach was used for other sections of the guidelines. The Grading of Recommendations, Assessment, Development and Evaluations (GRADE) methodology^12^ was adopted, similar to other EAACI guidelines.^13^ The expert group met periodically over a 2‐year period to appraise the results of the systematic reviews and to discuss the recommendations that were drafted in advance of video meetings. Recommendations were voted on electronically in real time using the Zoom voting platform (https://zoom.us/) managed by an independent member of the EAACI headquarters team. A minimum of 80% of votes from the expert group in favour of the recommendations was required for the recommendations to be approved.

### 2.4 Peer‐review and public consultation

These guidelines have been reviewed by the aforementioned expert group that formed the EAACI Task Force and by the EAACI Executive Committee. The guidelines were also submitted to public consultation through display on the EAACI website for 2 weeks and all feedback was carefully considered by the steering committee and incorporated, to the greatest extent possible, in the final version, which was reviewed and approved by all listed authors.

### 2.5 Conflicts of interest management

The EAACI Food Allergy Guidelines were commissioned and funded by EAACI to support the effort towards the systematic review of the literature and meta‐analyses. All members of the steering committee and of the expert group worked voluntarily without compensation and filled in a declaration of conflicts ahead of the start of the project, which were reviewed by EAACI.

## 3 GUIDELINE RECOMMENDATIONS

Table 1 summarises the recommendations about the management of IgE‐mediated food allergy, which are presented and discussed below.

**TABLE 1:** Recommendations about the management of IgE‐mediated food allergy.

Topic	Recommendations	Certainty of evidence	Strength of recommendation
Dietary management	In patients with confirmed IgE‐mediated food allergy, avoidance of the food (or form of the food) to which the patient is allergic is recommended.	Low^a^	Strong
	2 In patients with confirmed IgE‐mediated food allergy, continued consumption of tolerated foods is recommended.	Moderate^a^	Strong
	3 In patients with confirmed IgE‐mediated food allergy, age‐appropriate individualised dietary advice is suggested, with support from a registered dietitian for complex patients, if available.	Very low	Conditional
Psychological support	4 In selected patients with IgE‐mediated food allergy and their caregivers, psychological support by a trained health care professional is suggested.	Low	Conditional
Treatment plan	5 In patients with IgE‐mediated food allergy, an individualised management plan is recommended.	Low^a^	Strong
	6 In patients with IgE‐mediated food allergy at risk of anaphylaxis, prescription of adrenaline auto‐injectors to carry is recommended.	Low^a^	Strong
	7 In patients with IgE‐mediated food allergy at risk of anaphylaxis, structured, comprehensive training to improve recognition of anaphylaxis and use of adrenaline autoinjectors is recommended, in addition to basic instructions about autoinjector use.	Low^a^	Strong
Allergen‐specific immunotherapy (AIT)	8 In patients with IgE‐mediated food allergy eligible for allergen‐specific immunotherapy, its administration is recommended under the guidance of a clinical team with experience in food immunotherapy and in managing its side effects and anaphylaxis.	Low^a^	Strong
AIT for peanut allergy	9 In children and adolescents with IgE‐mediated peanut allergy, peanut oral immunotherapy is recommended to achieve desensitisation.	High	Strong
	10 In children and adolescents with IgE‐mediated peanut allergy, peanut epicutaneous immunotherapy is suggested to achieve desensitisation, if available.	High	Conditional
	11 In children and adolescents with IgE‐mediated peanut allergy, peanut sublingual immunotherapy is suggested to achieve desensitisation.	Moderate	Conditional
AIT for egg allergy	12 In children (generally above 4 years of age) and adolescents with IgE‐mediated egg allergy, egg oral immunotherapy is suggested to achieve desensitisation.	Low	Conditional
AIT for cow's milk allergy	13 In children (generally above 4 years of age) and adolescents with IgE‐mediated cow's milk allergy, milk oral immunotherapy is suggested to achieve desensitisation.	Low	Conditional
BIOLOGICS	14 In patients with IgE‐mediated food allergy, omalizumab is suggested to achieve desensitisation.	Moderate	Conditional

Abbreviation: AIT, allergen‐specific immunotherapy.

^a^ See text for justification of strength of recommendation given the certainty of evidence.

### 3.1 Dietary management

#### 3.1.1 Recommendation 1

In patients with confirmed IgE‐mediated food allergy, avoidance of the food (or form of the food) to which the patient is allergic is recommended.

##### Reason for recommendation

The complete avoidance of a known trigger food is the only option for many individuals with a confirmed IgE‐mediated food allergy to one or more foods.^14^ However, for food allergies such as pollen‐food syndrome, where it is known that cooked or processed versions of the food may be tolerated, then individuals may be able to only avoid the raw or unprocessed version of the trigger food.^15^ This is also true for children allergic to cow's milk or hen's egg where baked forms of the food can be tolerated even whilst still reacting to the fresh or raw form of the food.^16^, ^17^ For other foods, however, processing can increase allergenicity (e.g. roasting of nuts and seeds).^18^, ^19^, ^20^

##### Strength of recommendation

The evidence base is low as there have been no RCTs evaluating and comparing outcomes for patients with a food allergy who continue to eat the small amounts or an altered form of the food (e.g. baked) and those who completely avoid it—clinical trials are underway.^21^ However, the panel formulated a strong recommendation, placing a high value on the risk of a severe allergic reaction upon exposure to food that can be fatal.^22^, ^23^

##### Practical implications

Avoidance of a food can be problematic especially if the food is a staple food in the local diet or is a common ingredient (e.g. egg).^24^ Accidental exposure and reactions to trigger foods are common occurrences in individuals with food allergy.^25^ For this reason, all those with confirmed IgE‐mediated food allergy should receive age‐appropriate oral or written advice which is specific to the known food trigger(s), outlining which foods need to be avoided.^26^ In addition, major allergens (or the risk of cross‐contamination) should always be highlighted on the label by the producer or in the menu for non‐prepacked food.

#### 3.1.2 Recommendation 2

In patients with confirmed IgE‐mediated food allergy, continued consumption of tolerated foods is recommended.

##### Reason for recommendation

A nutritious and diverse diet is highly beneficial for all ages, but especially for growth, development, and the primary prevention of other allergic conditions in infants and children.^27^, ^28^ It is therefore important to ensure that, apart from the known trigger foods, all other foods should be introduced or re‐introduced into the diet and eaten regularly (i.e. weekly in an age‐appropriate portion size), including those foods to which the individual is sensitised but tolerant.^29^, ^30^ This applies also to processed forms of the food which are tolerated (e.g. baked milk and baked egg), whilst still avoiding less process form of the food (e.g. fresh milk and loosely cooked egg).

##### Strength of recommendation

There is moderate evidence to support the continued consumption of foods that are tolerated, and we placed high value on reducing delay in the introduction of complementary foods and possible nutritional deficit, so a strong recommendation was formulated.^4^, ^30^, ^31^, ^32^, ^33^, ^34^

##### Practical implications

Foods which have been previously tolerated, not linked to any allergic reaction and for which food allergy diagnosis has been appropriately excluded,^6^ should be reintroduced. Continued consumption of tolerated foods may need to be supported through counselling, and/or food challenges to confirm tolerance or remission, especially in individuals who are sensitised (ideally, patients should not be tested to foods that are tolerated but this can unfortunately happen prior to referral and lead to families avoiding the food). Foods related to an implicated allergen should not be automatically avoided and their consumption should be maintained (e.g. other tree nuts already tolerated in a hazel nut allergic child). In infants, there should be no delay to the introduction of other foods, especially peanut and egg (for which there is clear evidence of benefits of early introduction), if these are not one of the identified food triggers.^31^, ^35^, ^36^

#### 3.1.3 Recommendation 3

In patients with confirmed IgE‐mediated food allergy, age‐appropriate individualised dietary advice is suggested, with support from a registered dietitian for complex patients, if available.

##### Reason for recommendation

Individualised dietary advice is necessary to account for differences in traditional foods, cultural/religious dietary patterns, vegetarian or vegan diets, ethnic and racial differences and socio‐economic issues and to avoid nutritional deficiencies.^24^, ^37^, ^38^, ^39^, ^40^, ^41^ Such advice should focus on minimising or avoiding negative impact of food avoidance on nutritional intake, growth or developmental milestones, mental health and wellbeing of the individual, while reducing the risk of accidental exposure.^42^, ^43^ The provision of individualised dietary advice can support those who are excluding foods unnecessarily due to anxiety, or exhibiting selective eating behaviours, although more severe forms of food aversion such as Avoidant Restrictive Food Intake Disorder (ARFID) will require additional specialised psychological input.^44^, ^45^, ^46^, ^47^, ^48^, ^49^ Overly restrictive dietary practices reduce dietary diversity, can impact on food allergy prevention, mental health and the wellbeing of the individual and may encourage poor eating habits.^28^, ^50^, ^51^ It also imposes a significant cost burden on the patient and its family. Other allergic and non‐allergic co‐morbidities may also complicate the dietary management and affect food choice further.^52^, ^53^, ^54^, ^55^

##### Strength of recommendation

The evidence is very low, as there have been no studies evaluating whether actively providing individualised dietary advice on food exclusion compared to not giving any advice results in better outcomes for patients. It is also well recognised that there is a lack of specialist dietitians/nutritionists to provide high‐quality individualised dietary management.^56^ Therefore, a conditional recommendation was formulated. However, there is good evidence that individualised dietary counselling improves anthropometric and laboratory biomarkers of nutritional status in children with food allergy.^37^, ^57^, ^58^

##### Practical implications

Professional dietary management should encourage a healthy diet and provide options of what the allergic person can eat, not just what they cannot eat.^59^ Individuals of all ages should be given advice on allergen avoidance, foods free from the trigger allergen and suitable nutritional substitutes. Assessment of nutritional adequacy both at diagnosis and review should be undertaken irrespective of the food, to ensure dietary intake is not compromised due to the exclusion of one or more foods.^60^, ^61^ Management of infants should include regular review of weight, length and head circumference (<2 years), and dietary intake including vitamin/mineral supplementation, complementary feeding and dietary diversity, and support for breast feeding and any maternal dietary changes/elimination.^57^, ^59^, ^62^ This is especially pertinent to the management of cow's milk allergy which most commonly affects infants. Mothers of breast‐fed infants may need to exclude mammalian milks (or another culprit allergen) from their own diet (although this is rarely needed^63^), or if their baby is formula‐fed, be advised on suitable replacements. This is usually an extensively hydrolysed casein/whey formula, or hydrolysed rice formula as first line, with an amino acid formula utilised as a second option, as well as on the exclusion of other mammalian milks, and the age‐appropriate use of fortified plant‐based drinks.^64^, ^65^, ^66^, ^67^, ^68^

Dietary management should also include advice on the interpretation of food labels and “may contain” statements, which for composite dishes may pose a problem, safe and healthy eating at school/college/restaurants, managing food allergies when travelling/relocating to other countries, and how co‐factors (e.g. exercise, intercurrent illness, alcohol, pain relief, drugs) could affect the likelihood of an allergic reaction.^69^, ^70^, ^71^ Dietary management also needs to take into account economic issues, food insecurity, and cooking facilities.^72^, ^73^, ^74^, ^75^ Specialist ‘free from’ foods are usually more expensive and may have a lower nutritional value than standard food products so advice about alternative foods, recipes and practical help is often necessary.^75^ Dietary management should also support expansion of the diet when clinically indicated e.g. baked milk and/or egg, selective nut introduction or safe fruits and vegetables relating to pollen food syndrome.^15^, ^76^ Avoidance of co‐factors is suggested for patients tolerating certain forms of the allergen, such as patients with pollen‐food syndrome consuming the culprit fresh fruits and vegetables. The emphasis of dietary management should be on inclusion, rather than avoidance, and a personalised approach, rather than one size fits all.^77^

### 3.2 Psychological support

#### 3.2.1 Recommendation 4

In selected patients with IgE‐mediated food allergy and their caregivers, psychological support by a trained health care professional is suggested.

##### Reason for recommendation

Food allergies are often associated with heightened levels of anxiety, worry, and stress, significantly impacting the quality of life for both patients and their families.^45^, ^78^ A recent systematic review by Knibb et al.^11^ highlights the referral of patients and families for psychological support may reduce distress and improve food allergy management and quality of life. The authors suggest that cognitive‐behavioural based interventions facilitated by a healthcare professional are beneficial for patients with moderate to severe food allergy related distress. While access to specialised psychological support may be limited for some individuals with food allergies, healthcare providers can still play a vital role in addressing the psychological impact of the condition and connecting patients with available resources and support services. This approach can contribute to improved management of food allergies and enhanced quality of life for patients and their families.

##### Strength of recommendation

The certainty of evidence is low as number of studies is limited, there is heterogeneity in the interventions studied including education, peer mentoring, self‐regulation theory and other coping technics facilitated by a wide range of healthcare professionals.

##### Practical implications

Referral to a mental health or well‐being specialist or psychologist may not be readily available or feasible for all patients and families dealing with food allergies. Therefore, it is important to prioritise access to these services for those experiencing moderate to severe distress related to their condition. For individuals with milder forms of anxiety or stress related to food allergies, there may be alternative resources available within the healthcare system. This could include access to online or in person support groups, remote guidance from HCP, charities or third sector organisations, educational materials, or counseling sessions facilitated by healthcare professionals with training in allergy management and psychological support. Psychological support may not just be one‐time, but encompass dietary management to immunomodulatory treatment and regular assessment may be needed.

### 3.3 Treatment plan

#### 3.3.1 Recommendation 5

In patients with IgE‐mediated food allergy, an individualised management plan is recommended.

##### Reason for recommendation

IgE‐mediated food allergic reactions can range from mild to severe (i.e. anaphylaxis). Patients should be empowered to identify allergic reactions and commence appropriate treatment and emergency care. Patient‐specific medical treatment plans facilitate recognising and treating allergic reactions and communication with emergency services.

##### Strength of recommendation

The evidence is low, since controlled trials comparing the issuing of an individualised treatment plan versus a generic plan have not been performed. The strength of recommendation is strong, as there is evidence that the early recognition and treatment of allergic symptoms may improve outcomes and that a plan may facilitate this process.

##### Practical implications

Healthcare practitioners should provide individualised treatment plans as part of the standard care package for food allergic patients. Patients should be educated to recognise allergic symptoms and what they mean and understand which medications should be administered in response. The plan must detail the contact information for local medical services to expedite acute medical care. Treatment plans should be age‐appropriate, have clear stepwise instructions and be written in a language understood by the patient and responders in the specific geographic and cultural setting. Treatment plans should be patient‐specific, matching the patient's particular list of food allergens to be avoided and medications to be taken, which can include oral non‐sedative anti‐histamine, inhaled salbutamol and adrenaline auto‐injector (AAI), depending on the patients' risk profile. The treatment plan should detail how to administer medications that are included in the plan. If antihistamines are included, these should be recommended in a form that the patient can swallow (younger children may not be able to swallow tablets), and families should know how to correctly administer inhaler medications through a spacer and mask. If an AAI is included in the plan, then training in the correct administration will also be required. The treatment plan should be available in a format that best suits the patient, such as a printed copy saved and saved on electronic platforms.

#### 3.3.2 Recommendation 6

In patients with IgE‐mediated food allergy at risk of anaphylaxis, prescription of AAI to carry is recommended.

##### Reason for recommendation

IgE‐mediated allergic reactions can result in life‐threatening reactions (anaphylaxis) for which adrenaline is the drug of choice for treatment. The WHO lists adrenaline as an essential medicine. In anaphylaxis, adrenaline should ideally be administered intramuscularly, using a preloaded AAI with an adequate needle length and a dosage adapted to weight and age. Adrenaline administration using an AAI is faster than administered with a syringe and needle and allows for more accurate dosage.

##### Strength of recommendation

The certainty of evidence is low as studies that compare the provision of adrenaline AAI to patients at risk of anaphylaxis versus not doing so in the real‐world acute setting are lacking. However, the panel formulated a strong recommendation, as it places a high value on the benefit–risk ratio, ethical considerations and on evidence from studies in non‐acute research settings.

##### Practical implications

Healthcare professionals should identify patients with IgE‐mediated food allergy at risk of anaphylaxis and prescribe an AAI. Table 2 summarises the indications for prescribing AAI for IgE‐mediated food allergy, as recommended in the EAACI Anaphylaxis Guidelines.^13^ Patients and caregivers must be empowered to identify anaphylaxis and correctly administer adrenaline, preferably using an AAI device. The European Medicines Agency and the Medicines and Healthcare products Regulatory Agency (MHRA) recommends that patients have access to two devices – see EAACI Anaphylaxis Guidelines^13^ for arguments for prescribing one versus two AAI devices. In some parts of the world, AAI is unfortunately not available; in this case, education and training of patients and families in the use of adrenaline vials and syringe may be required. Intra‐nasal adrenaline has recently received regulatory approval. Other modes of adrenaline administration are in various stages of development and may become available in the future.

**TABLE 2:** Indications for prescribing AAI in IgE‐mediated food allergy, according to the current EAACI Anaphylaxis Guidelines.^13^

Absolute indications	Relative indications
Previous episodes of food‐induced anaphylaxis Co‐existing unstable or moderate to severe persistent asthma Underlying systemic mastocytosis	Allergic reaction to foods known to be associated with anaphylaxis Allergic reaction to trace amounts of food Teenager or young adult Remote from medical help or prolonged travel abroad Patients on allergen‐specific immunotherapy

#### 3.3.3 Recommendation 7

In patients with IgE‐mediated food allergy at risk of anaphylaxis, structured, comprehensive training to improve recognition of anaphylaxis and use of adrenaline autoinjectors is recommended, in addition to basic instructions about autoinjector use.

##### Reason for recommendation

Anaphylaxis is a potentially life‐threatening allergic reaction for which adrenaline remains the treatment of choice. In the community, adrenaline should ideally be administered intramuscularly using an AAI, with an adequate needle length and a dosage adapted to weight and to age.

##### Strength of recommendation

There are data to support different training strategies for recognising anaphylaxis and its treatment; however, they were not obtained in an acute real‐world setting. Therefore, the certainty of evidence is low, but the strength or recommendation is strong as it places high value on the patients' safety.

##### Practical implications

Training programs for patients and caregivers that focus on recognising anaphylaxis and other allergic symptoms and signs and stepwise treatment are essential. Training teachers and peers is also important. A switch of focus from child to adolescent and in transition to adulthood is key to ensure that patients feel confident and empowered to manage their allergic reactions and self‐administer treatment, if needed. Training programs should be tailored to the specific patient setting, age, language, and baseline allergy understanding of the disease and risk of anaphylaxis. Different strategies and educational tools may be required, sometimes repeatedly, to empower patients to identify allergic symptoms and initiate medical treatment. Indeed, AAI training should be repeated as part of every follow up visit.

Patients and their caregivers must be empowered to identify and treat anaphylaxis. Whilst definitions of anaphylaxis vary across the world, all these reactions are severe and life‐threatening. We shall refer to the EAACI definition of anaphylaxis as a reference.^13^ It is critical that patients can identify airway, breathing and circulatory compromise as these can prove fatal. Skin and mucosal changes are usually present but are not required to support a diagnosis of anaphylaxis.

Adrenaline administration, preferably using an AAI device, remains the treatment of choice in moderate–severe food‐induced allergic reactions. AAI are available in many countries worldwide, but access and reimbursement greatly vary. Doses may include 0.15 mg, 0.3 mg and 0.5 mg adrenaline (additionally, 0.1 mg in the US), and the prescribing clinician should best match these to the patient's age and weight. The instructions for each device vary, so patients must be trained to use the device they are carrying. Patients must be trained to identify the correct body positioning during anaphylaxis and the anatomical landmarks needed for AAI administration into the thigh muscle.

### 3.4 Allergen‐specific immunotherapy

#### 3.4.1 Recommendation 8

In patients with IgE‐mediated food allergy eligible for allergen‐specific immunotherapy, its administration is recommended under the guidance of a clinical team with experience in food immunotherapy and in managing its side effects and anaphylaxis.

##### Reason for recommendation

Allergen‐specific immunotherapy (AIT) consists in the administration of allergen to allergic patients and, therefore, has the potential for inducing allergic reactions, of variable and unpredictable severity. Although AIT is generally deemed to be safe in our recent systematic review,^9^ an earlier systematic review by Chu et al^79^ reported, for example, that patients on peanut oral immunotherapy (POIT) have a higher rate of allergic reactions to peanut compared with peanut allergic patients on a standard allergen avoidance diet, which may arguably not be the ideal comparator. The risk of allergic reactions and anaphylaxis is greater at the start of treatment and up‐dosing. Thus, these stages should be performed in hospital or in the office, only if appropriate resources, equipment and experienced personnel are available to treat allergic reactions of any degree of severity, including anaphylaxis.^13^ The clinical team delivering AIT should be specifically trained in this procedure, have standardised protocols and plans for treatment adjustment, if necessary, in the event of an adverse reaction. Patient safety is paramount.

##### Strength of recommendation

Evidence from a previous systematic review of the risk of allergic reactions during AIT^79^ makes close monitoring during AIT by an experienced team a strong recommendation.

##### Practical implications

Allergen‐specific immunotherapy should be offered to patients with confirmed IgE‐mediated allergy to that food, for whom benefits outweigh the risks. Studies of AIT (via oral, sublingual and epicutaneous routes) in pre‐school age children have shown greater efficacy in terms of desensitisation and remission compared to studies in older children and adults.^80^, ^81^, ^82^, ^83^, ^84^, ^85^ Initiation and up dosing of AIT (especially oral immunotherapy and sublingual immunotherapy) should be conducted in a clinical setting with resources to recognise and treat anaphylaxis by a clinical team that has the specialist training and expertise to deliver this treatment. The recommendation is not limited to regulatory‐approved products. Adrenaline auto‐injector should be prescribed to patients undergoing AIT. Education about allergic reactions and their management is essential. Avoidance of co‐factors during dosing is recommended. Like for other forms of AIT, families should be informed and shared decision making is encouraged to determine whether this is the right treatment for the patient – this depends not only on efficacy in terms of desensitisation but also on the long‐term expectations about food consumption (whether there is the desire to consume the food or simply to be protected from accidental exposure), effect of daily treatment and need to avoid co‐factors on the life‐style and management of risk of allergic reactions by patients and caregivers.

### 3.5 Peanut‐specific immunotherapy

#### 3.5.1 Recommendation 9

In children and adolescents with IgE‐mediated peanut allergy, peanut oral immunotherapy is recommended to achieve desensitisation.

##### Reason for recommendation

Peanut allergy and allergen avoidance can have a significant negative impact on the lives of patients and families. Anxiety, various social restrictions, and reduced quality of life have been reported.^86^, ^87^, ^88^ POIT can increase the threshold of reactivity in allergic patients and reduce the risk of accidental exposure, while patients are on treatment.^9^ The evidence for long‐term tolerance or remission of peanut allergy with POIT is yet to be demonstrated. A product for POIT has secured regulatory approval by EMA, MHRA and FDA, and is commercially available in some countries in Europe, the UK and the USA.

##### Strength of recommendation

Overall, our systematic review provided moderate quality of the evidence regarding the use of POIT to induce desensitisation.^9^ For instance, the relative risk (RR) for POIT was 11.94 (95% CI 1.76–80.84) compared to avoidance or placebo. However, considering only the low‐risk‐of‐bias studies, the RR for desensitisation with POIT was 49.98 (95% CI 7.04–355.2) compared to avoidance and 27.36 (95% CI 6.97–107.38) compared to placebo.^9^ In the sub‐analyses by age group, the RR was 35 (95% CI 5.07–247.57) in children younger than 4 years and 8.23 (95% CI 3.59–18.86) in children aged 4–17 years compared to placebo.^9^ For the youngest age group, key studies were published after completion of the systematic review and meta‐analyses and are summarised in Table 3, together with the seminal study by Vickery et al. from 2017. The high certainty of evidence of desensitisation achieved with POIT in children and adolescents supported a strong recommendation. There was limited evidence of efficacy of POIT in adults; thus, no recommendation could be made for this age group.

**TABLE 3:** Summary of studies of peanut immunotherapy in children under 5 years of age and Outmatch study published since completion of the systematic review and meta‐analyses that informed these guidelines.^80^, ^81^, ^82^, ^83^, ^84^, ^89^, ^90^

Study	Design	Intervention	Age	Number of patients included	Efficacy	Safety
Vickery et al 2017^80^	Randomized (no placebo)	OIT peanut Primary end point, SU at 4 weeks after stopping early intervention oral immunotherapy either upon achieving 4 prespecified criteria, or after 3 maintenance years. Maintenance doses of 300 or 3000 mg/day	9 to ≤36 months	37	29 of 37 (78%) in the intent‐to‐treat analysis achieved 4‐SU (300‐mg arm, 17 of 20 [85%]; 3000 mg, 12 of 17 [71%], *p* = .43) over a median of 29 months. Per‐protocol, the overall proportion achieving 4‐SU was 29 of 32 (91%)	Mild to moderate
Loke et al 2022^81^	RCT	Probiotic and peanut oral immunotherapy. Were followed up until 12 months after completion of treatment. Dual primary outcomes were 8‐week SU	12 to ≤60 months (Overall study group included children until 10 years of age)	104	36 (46%) of 79 children in the PPOIT group and 42 (51%) of 83 children in the OIT group achieved SU compared with two (5%) of 39 children in the placebo group (risk difference 40·44% [95% CI 27·46 to 53·42] for PPOIT vs. placebo, *p* < .0001), with no difference between PPOIT and OIT (−5.03% [−20.40 to 10.34], *p* = .52)^a^	Treatment‐related adverse events were reported in 72 (91%) of 79 children in the PPOIT group, 73 (88%) of 83 children in the OIT group, and 28 (72%) of 39 children in the placebo group Addition of a probiotic might offer a safety benefit compared with OIT alone, particularly in preschool children^a^
Jones et al 2022^82^	RCT	OIT peanut (2000 mg peanut protein per day) Primary outcome was desensitisation at the end of treatment (week 134), and remission after avoidance (week 160)	12 to ≤48 months	146	At week 134, 68 (71%, 95% CI 61–80) of 96 participants who received peanut OIT compared with one (2%, 0.05–11) of 50 who received placebo met the primary outcome of desensitisation (risk difference [RD] 69%, 95% CI 59–79; *p* < .0001) After avoidance, 20 (21%, 95% CI 13–30) of 96 participants receiving peanut OIT compared with one (2%, 0.05–11) of 50 receiving placebo met remission criteria (RD 19%, 95% CI 10–28; *p* = .0021)	Most participants (98% with peanut OIT vs. 80% with placebo) had at least one OIT dosing reaction, predominantly mild to moderate and occurring more frequently in participants receiving peanut OIT. 35 OIT dosing events with moderate symptoms were treated with adrenaline in 21 participants receiving peanut OIT
Du Toit et al 2023^83^	RCT	OIT peanut The primary end point was desensitization (i.e. tolerating a ≥600 mg single dose of peanut protein with only mild allergy symptoms)	12 to ≤48 months	146	In the treated group (*n* = 98), 73.5% of participants tolerated a single dose of ≥600 mg peanut protein at exit DBPCFC compared with 6.3% in the placebo group (*n* = 48)	Most participants experienced an adverse event (98.0% of OIT treated and 97.9% of placebo participants), which was mild or moderate in grade for 93.2% of participants (92.9% in OIT treated and 93.8% in placebo‐treated participants). Treatment‐related adverse events, which were mild to moderate, were experienced by 75.5% of OIT treated and 58.3% of placebo participants. Three treatment‐related systemic allergic reactions, none of which were severe or serious in grade, were noted in two OIT treated participants (2%)
Greenhawt et al. 2023^84^	RCT	EPIT peanut The primary end point was a treatment response as measured by the eliciting dose of peanut protein at 12 months	12 to ≤36 months	362	The primary efficacy end point result was observed in 67.0% of children in the intervention group as compared with 33.5% of those in the placebo group (risk difference, 33.4 percentage points; 95% confidence interval, 22.4 to 44.5; *p* < .001)	Adverse events that occurred during the use of the intervention or placebo, irrespective of relatedness, were observed in 100% of the patients in the intervention group and 99.2% in the placebo group. Serious adverse events occurred in 8.6% of the patients in the intervention group and 2.5% of those in the placebo group; anaphylaxis occurred in 7.8% and 3.4%, respectively. Serious treatment‐related adverse events occurred in 0.4% of patients in the intervention group and none in the placebo group. Treatment‐related anaphylaxis occurred in 1.6% in the intervention group and none in the placebo group
Kim et al. 2024^85^	RCT	SLIT peanut Desensitization was assessed by DBPCFC after 36 months of treatment. Participants desensitized to at least 443 mg peanut protein discontinued therapy for 3 months and then underwent DBPCFC to assess for SU	12 to ≤48 months (overall study group included children until 10 years of age)	50	The primary end point of desensitization was met with actively treated versus placebo participants having a significantly greater median cumulative tolerated dose (4443 mg vs. 143 mg), higher likelihood of passing the month 36 DBPCFC (60% vs. 0%), and higher likelihood of demonstrating SU (48% vs. 0%)	Oropharyngeal itching was more commonly reported by peanut SLIT than placebo participants. Skin, gastrointestinal, upper respiratory, lower respiratory, and multisystem adverse events were similar between treatment groups
Wood et al. 2024^91^	RCT	Omalizumab as monotherapy in patients with multiple food allergies The primary end point was ingestion of peanut protein in a single dose of 600 mg or more without dose‐limiting symptoms. The three key secondary end points were the consumption of cashew, of milk, and of egg in single doses of at least 1000 mg each without dose‐limiting symptoms	1 to 55 years of age The analysis population consisted of the 177 children and adolescents (1 to 17 years of age)		A total of 79 of the 118 participants (67%) receiving omalizumab met the primary end‐point criteria, as compared with 4 of the 59 participants (7%) receiving placebo (*p* < .001). Results for the key secondary end points were consistent with those of the primary end point (cashew, 41% vs. 3%; milk, 66% vs. 10%; egg, 67% vs. 0%; *p* < .001 for all comparisons)	Safety end points did not differ between the groups, aside from more injection‐site reactions in the omalizumab group

*Note*: DBPCFC, double‐blind placebo‐controlled food challenge; EPIT, epicutaneous immunotherapy; OIT, oral immunotherapy; RCT, randomised controlled trial; SLIt, sublingual immunotherapy; SU, sustained unresponsiveness; SCD, successfully consumed dose.

^a^ Note that efficacy and safety results are given for the overall patient groups (all ages), not specified by age group.

##### Practical implications

Following confirmation of peanut allergy diagnosis (see EAACI guidelines on diagnosis of IgE‐mediated food allergy^6^), children and adolescents (i.e. patients aged less than 18 years) should be offered POIT to increase their threshold of reactivity while on treatment. After completion of treatment, transition from AIT to real‐world peanut is allowed to enable regular peanut consumption and maintenance of therapeutic effect.^92^ Treated patients should be required to carry the AAI and other rescue medications, such as oral antihistamine and/or salbutamol inhaler, as appropriate. Patients and families should be carefully instructed and trained on how to recognise and treat allergic reactions and anaphylaxis. Recommendations to stop or reduce dosing during infections should be tailored to individual patients and advice on restricting co‐factors, such as exercise, hot showers, NSAIDs, before and after POIT dosing is advised and should be tailored to the individual patient.

#### 3.5.2 Recommendation 10

In children and adolescents with IgE‐mediated peanut allergy, peanut epicutaneous immunotherapy is suggested to achieve desensitisation, if available.

##### Reason for recommendation

Given the common side‐effects of OIT^79^ and its intolerability for some patients (e.g. due to gastrointestinal symptoms), other treatment modalities that involve the administration of lower allergen doses, through different routes, such as the epicutaneous route, have been explored. The heterogeneity in efficacy outcomes between trials hampers direct comparisons; however, generally the peanut tolerated dose with epicutaneous immunotherapy (EPIT) are lower than those achieved with OIT. EPIT has shown to increase the threshold of reactivity of peanut allergic patients and to protect from accidental exposure to commonly encountered allergen doses.^9^ EPIT was well‐tolerated and side‐effects were limited to the skin, namely to the site of application of the device, in most patients; however, anaphylactic reactions can occur.^9^

##### Strength of recommendation

The quality of evidence supporting the use of EPIT in peanut allergy was moderate,^9^ with 2.17 (95% CI 1.53–3.09) RR for inducing desensitisation compared with placebo—the three studies included in the meta‐analyses all had low risk‐of‐bias. Like in peanut OIT, a study focusing on the under‐five age group showed that EPIT is efficacious and safe^84^ (Table 3). However, there is no regulatory approved EPIT product available. As the patch is a specific device that cannot be replaced by real‐world peanut, a conditional recommendation was formulated to reflect the current unavailability of this treatment outside of research trials.

##### Practical implications

Once a patch for peanut EPIT is approved by regulatory authorities and available to clinicians, peanut EPIT can be used to induce desensitisation to peanut. Given its superior safety profile, it may be more suited to highly sensitive patients, patients with known gastrointestinal problems, highly anxious patients or patients who do not tolerate side effects or cannot accept the life‐style restrictions imposed by peanut OIT. EPIT may be followed by peanut OIT, if needed, or real‐world peanut, once treatment is completed, to enable achievement of higher peanut allergen doses and continuing peanut consumption, respectively. Treated patients need to continue carrying their rescue medication and written treatment plan and should be informed about possible side effects and their appropriate management.

#### 3.5.3 Recommendation 11

In children and adolescents with IgE‐mediated peanut allergy, sublingual immunotherapy (SLIT) is suggested to achieve desensitisation.

##### Reason for recommendation

SLIT has shown efficacy in inducing desensitisation (RR = 3.0, 95% CI 1.04–8.66 compared to placebo^9^) with allergen doses that are intermediate between those used in OIT and EPIT. The safety profile is very good with most adverse reactions limited to the oral cavity, with itchiness, discomfort, and angioedema. A recent publication by Kim et al.^85^ (Table 3) reported high efficacy and tolerability in 1–4‐year olds, which is the age group with the greatest potential for immunomodulation to induce long‐term remission, as for peanut OIT and EPIT.

##### Strength of recommendation

In the systematic review of the literature and meta‐analyses informing this guideline, we concluded the quality of evidence to be low; however, the recent publication by Kim et al,^85^ increased the evidence level to moderate. Like EPIT, there is no commercially available regulatory approved product for peanut SLIT. Therefore, the panel formulated a conditional recommendation.

##### Practical implications

Once a standardised product is available for peanut SLIT, this can be used. A standardised regulatory‐approved SLIT product would probably be more amenable to use for SLIT than the actual food. However, it is important to document the protein quantity and allergenicity used for treatment and to use a standardised product, not only for research and clinical trials, but also for a rigorous application of this treatment to routine clinical practice.

### 3.6 Egg‐specific immunotherapy

#### 3.6.1 Recommendation 12

In children (generally above 4 years of age) and adolescents with IgE‐mediated egg allergy, egg oral immunotherapy is suggested to achieve desensitisation.

##### Reason for recommendation

Unlike peanut allergy, which tends to be persistent in ca 70%–80% of children, egg allergy spontaneously resolves in 50%–60% of allergic children by school age.^93^, ^94^, ^95^ This fact, together with the costs and time associated with OIT, led to recommending egg OIT generally from 4 years of age. The expert group recognised that there is a more severe phenotype of egg allergy, that tends to be more persistent^96^ and may be recognised prior to the age of 4 years; thus, did not restrict egg OIT to children above this age but rather use this age as an indicator that egg allergy may resolve and it is in children with persistent egg allergy that OIT is most warranted. This is further supported by the evidence synthesised in our meta‐analyses. Egg OIT had 3.43 (95% CI 2.24–5.27) RR overall, with 3.99 (95% CI 2.55–6.25) compared to avoidance and 2.22 (95% CI 0.96–5.06) compared to placebo. However, in the sub‐analyses by age group, only the 4‐17‐year group (and not the under 4 s) had a significant RR for desensitisation with egg SLIT. The subgroup 12‐17‐years also did not show a significant RR. In the sub‐analyses of low‐risk‐of‐bias studies, the RR is 5.57 (95% CI 0.96–32.27) was not statistically significant. No data is available for adults or SLIT or EPIT for egg allergy, thus no recommendation could be made regarding these age group or routes of administration. There was also no evidence for OIT using different degrees of processing of egg (e.g. baked, cooked, raw egg); however, the difference in allergenicity could have implications in both efficacy and safety.

##### Strength of recommendation

The quality of evidence supporting the use of egg OIT for desensitisation in children older than 4 years was overall low. This, together with the fact that there is no standardised regulatory‐approved product available, led to a conditional recommendation being made.

##### Practical implications

In children with confirmed egg allergy, particularly if older than 4 years and/or considered to have persistent egg allergy, egg OIT can be offered using the actual food. In the future, should a standardised product be approved by regulators, this would be preferred. However, with the potential higher cost and reduced accessibility to patients. Like for peanut OIT, avoidance of co‐factors should be advised and tailored to the individual patient.

### 3.7 Cow's milk‐specific immunotherapy

#### 3.7.1 Recommendation 13

In children (generally above 4 years of age) and adolescents with IgE‐mediated cow's milk allergy, milk oral immunotherapy is suggested to achieve desensitisation.

##### Reason for recommendation

Similar to egg allergy, cow's milk allergy is often outgrown, thus the recommendation included patients aged 4 years or older and excluded adults, for whom there is no evidence. The milk OIT studies were all at highrisk‐of‐bias and thus low risk‐of‐bias‐only meta‐analyses were not possible. There was statistically significant difference of milk OIT compared to avoidance (RR = 6.31, 95% CI 1.91–20.83) and to soya milk (RR = 21, 95% CI 1.34–328.86) but not compared to placebo (RR = 4.61, 95% CI 0.02–1253.17).^9^ These trends were also observed in the sub‐analyses for children and adolescents aged 4–17 years. The sub‐analyses for children younger than 4 years included only one study, which compared milk OIT with avoidance and showed a significant RR of 3.86 (95% CI 1.99–7.46).^9^

##### Strength of recommendation

The quality of evidence supporting milk OIT was considered low and thus the recommendation was conditional.

##### Practical implications

Milk OIT is a treatment option for children with persistent milk allergy, and thus should be considered after a period of follow‐up to see whether spontaneous resolution of the allergy occurs. Like for egg, this is not a strict timeline and OIT can be started in younger children if considered beneficial. There is no regulatory approved product in the market and often fresh cow's milk is used for milk OIT. Like for peanut and egg OIT, avoidance of co‐factors should be advised and tailored to the individual patient undergoing milk OIT.

### 3.8 Biological treatments

#### 3.8.1 Recommendation 14

In patients with IgE‐mediated food allergy, omalizumab is suggested to achieve desensitisation.

##### Reason for recommendation

Our meta‐analysis showed a positive effect of omalizumab in terms of desensitisation to the culprit food (RR = 2.17, 95% CI 1.22–3.85), but the RR was not significant for long‐term effect.^9^ Sub‐group analyses restricted to low‐risk‐of‐bias studies supported the use of omalizumab to treat IgE‐mediated food allergy. Small observational studies suggest that a significant portion of avoided foods can be reintroduced during treatment with omalizumab without the need for any oral immunotherapy.^97^ The primary outcome of the Outmatch study reported on the effect of omalizumab as monotherapy on the threshold of reactivity to peanut and other foods commonly implicated in food allergy^91^ (namely, cashew nut, egg, and milk). Most (67%) patients treated with omalizumab tolerated at least a single dose of 600 mg of peanut protein without dose‐limiting symptoms compared to 7% of placebo‐treated subjects, and with other foods, at least a single dose of 1000 mg of protein was tolerated without dose‐limiting symptoms in 41% versus 3% to cashew nut, 66% versus 10% to cow's milk and 67% versus 0% to egg. Adverse events were similar between study arms. As a result of these new data, omalizumab was approved by FDA and licensed for treatment of IgE‐mediated food allergy in the US, in children as young as 1 year of age. However, neither omalizumab nor other biologics are currently licensed for IgE‐mediated food allergy in any other part of the world.

##### Strength of recommendation

The quality of evidence considering the studies included in the systematic review and meta‐analyses was moderate, and most studies had a highrisk‐of‐bias.^9^ The results of the Outmatch study strengthen the evidence in support of the use of omalizumab to treat IgE‐mediated food allergy.^91^ Despite the high‐quality evidence, omalizumab is only currently licensed for use in food allergy in the USA; therefore, the recommendation is conditional.

##### Practical implications

Omalizumab can be used to manage IgE‐mediated food allergy, if available. Omalizumab is only approved in the USA currently and is an expensive treatment. Current omalizumab license foresees avoidance of the culprit food in the diet and continued carriage of adrenaline auto‐injector and other medications specified in the treatment plan. The continued need for allergen avoidance may limit patients' quality of life. The long‐term efficacy of omalizumab is unknown, especially in the absence of food introduction or AIT, and its effect should be expected to be lost after discontinuation of treatment.

## 4 DISCUSSION

### 4.1 Summary

Food allergy can have an immense negative impact on the day‐to‐day life of patients and their families. An accurate precise diagnosis is of the essence and a disease‐modifying treatment is desirable. In all cases, appropriate dietary management, including avoidance of culprit foods and continued consumption of tolerated safe foods, is extremely important, ideally under the advice of a specialised dietitian. In addition, prescription of emergency medication for treatment of allergic reactions resulting from accidental exposure is necessary – this can include AAI for intramuscular injection, oral antihistamine and inhaled salbutamol. Patients with anxiety needing support for coping with impact of food allergy on their lifestyle may benefit from the referral to a clinical psychologist. In terms of immunomodulatory treatments, omalizumab has been approved in the USA for management of IgE‐mediated food allergy in children from 1 year of age and adults; and oral allergen‐specific immunotherapy can be recommended for peanut, egg and milk allergies. For peanut allergy, sublingual and epicutaneous immunotherapy are suggested but are not yet available in most clinical settings.

### 4.2 Strengths and limitations

These IgE‐mediated food allergy management guidelines are evidence‐based and follow the GRADE methodology. They were rigorously developed by a large group of scientific and clinical experts, members of the multidisciplinary allergy team and patient representatives from Europe and beyond. They were informed by a dedicated systematic review of the literature that was conducted by an independent research team under the guidance of the steering committee.

The main limitations are the quality of the evidence available, with most studies having highrisk‐of‐bias, and the heterogeneity in methodology and outcomes of published studies. There is a pressing need for more studies, covering more treatment modalities and more food allergies, with a standardised methodology and outcomes that are tailored for clinical practice and the food allergic patients that are seen around the world. For instance, the evidence on immunotherapy to tree nuts, sesame, peach and other foods is limited, thus, no recommendations could be made about immunotherapy to foods other than peanut, egg and cow's milk. As an example, in some parts of Europe, lipid‐transfer protein (LTP) syndrome due to allergy to lipid transfer proteins from various foods is the most common cause of allergy to plant foods (fruits, vegetables and nuts) and a major cause of severe anaphylaxis. Immunotherapy with peach extract enriched for LTP has been commercially available in some countries and there are some publications of clinical trials and real‐life studies. Although these studies were captured in the systematic review that informed these guidelines,^98^, ^99^ it was not possible to conduct meta‐analyses nor to make any recommendations about immunotherapy to LTP.

Another important limitation is our inability to include new studies that have been published since the systematic review was concluded,^80^, ^85^ including the study that informed the approval of omalizumab in the USA and the peanut immunotherapy studies in children younger than 5 years that demonstrated impressive efficacy. These studies were, however, considered in the expert discussions that led to the elaboration of the recommendations published here.

Table 4 lists gaps in the evidence and resulting research needs. The next stage, following the publication of the guidelines, is their implementation in clinical practice. Table 5 lists the barriers and facilitators of implementation of these guidelines.

**TABLE 4:** Gaps in the evidence and research needs in the management of IgE‐mediated food allergy.

Gaps in the evidence	Research need	Priority
Additional effect of early introduction to prevent allergy in children who already are food allergic	Review of prevalence of new‐onset allergies in children who already have an allergy to a food, and the role of diet diversity in preventing additional food allergies in those with current food allergies A better understanding of optimal age of introduction in different at‐risk groups and assessing the optimal dosing schedule for introduction in terms of quantity of food and frequency/duration of exposure	HIGH
Efficacy and safety of low‐dose allergen consumption in patients with high threshold of reactivity	Clinical trials for treatment of allergic patients with high threshold of reactivity	HIGH
Efficacy and safety data on (oral, sublingual and epicutaneous) immunotherapy to foods other than peanut	High‐quality clinical trials of (oral, sublingual and epicutaneous) immunotherapy for foods other than peanut	HIGH
Efficacy and safety data on biologics for food allergy other than omalizumab	High‐quality clinical trials of biologics for food allergy, including new therapeutic targets	HIGH
Efficacy of different dosing regimens of Omalizumab (low‐dose, less frequent, weight‐based dosing), optimal duration of treatment and effects post‐discontinuation low‐dose	RCT of different dose regimens, frequency and duration of treatment in children and adults	HIGH
Additional effect of early introduction to prevent allergy in children who already are food allergic	Review of prevalence of new‐onset allergies in children who already have an allergy to a food, and the role of diet diversity in preventing additional food allergies in those with current food allergies A better understanding of optimal age of introduction in different at‐risk groups and assessing the optimal dosing schedule for introduction in terms of quantity of food and frequency/duration of exposure	HIGH
Efficacy and safety of low‐dose allergen consumption in patients with high threshold of reactivity	Clinical trials for treatment of allergic patients with high threshold of reactivity	HIGH
Assessing long term tolerance in patients undergoing food immunotherapy.	High quality data for long term follow‐up including safety profile, quality of life and health economics.	HIGH
Safety and efficacy OIT versus SLIT versus EPIT	Head‐to‐head studies addressing different routes and outcomes in relation to desensitization and quality of life.	HIGH
Safety and efficacy Multifood OIT	High quality trials defining Multifood OIT, its optimal protocol and assessing safety with and without biologics.	HIGH
The effect of individualised dietary management regarding accidental exposure	Prospective assessment of allergic individuals on accidental exposure Comparison of providing individual dietary advice by a registered dietitian compared with giving generic advice to see if this reduces accidental exposure.	MEDIUM
Phenotypic characterisation/risk factors affecting nutritional adequacy in patients with an already restricted diet	The nutritional status of those individuals with a diagnosed food allergy who are already on a plant‐based diet or excluding wheat/other grains.	MEDIUM
The effect of ethnic and socio‐economic factors, on nutritional intake, accidental exposure and allergy resolution	Reviews of socio‐economic status and ethnicity of patients with food allergy in Europe.	MEDIUM
Efficacy and safety of combination of different biologics for treatment of IgE‐mediated food allergy	RCT of combination of biologics	MEDIUM
Understanding the most effective psychological interventions available for patients with food allergy how it should be carried out	High‐quality clinical trials comparing head‐to‐head psychological interventions and their outcomes	MEDIUM
Assessing the patient profile which will benefit from diverse psychological and mental health interventions related to food allergy anxiety and quality of life	Clinical trials assessing the patient profile who will benefit from diverse psychological interventions	MEDIUM

**TABLE 5:** Implementation of the EAACI guidelines on management of IgE‐mediated food allergy.

Area	Barriers to implementation	Facilitators to implementation	Audit criteria	Resource implications
Dietary management	Insufficient or no specialist allergy dietitians and/or nutritionists, including clinicians to provide support to enable dietary management of introduction of foods including in the community Unfiltered information from online sources Over‐avoidance of foods due to fear of further allergies, or because of incorrect advice on blanket elimination	More integration of specialised dietitians and/or nutritionists as part of the allergy team and recognition of the importance of specialist dietary management Patients/parents becoming increasingly interested in nutrition/diet More widespread availability of allergen alternatives Online resources (EAACI patient information video on dietary diversity, Allergy UK weaning pack, food refusal, selective eating and feeding difficulties website)	Number of patients who have access to individualised dietary management	Need for food allergy trained dieticians
Treatment plan	Insufficient or no access to allergy clinics and HCP's skilled in the management of allergy Treatment plans that are not age, language, region, country appropriate Digital Poverty Restricted access to plans in electronic format. Unfiltered information from online sources	Training of HCP's in the importance of issuing individualised treatment plans. Recognition of the importance of treatment plans that are age, language, region, country specific Patients/parents/carers recognising the importance of treatment plans Structured information from online sources	Number of patients who have received a personalised treatment plan after diagnosis Number of patients/families/carers who are able to appropriately act on the step‐wise advice in the treatment plan.	Training of HCP Cost associated with staff resources required to issue and train the patient/family and caregivers in the use of the treatment plan. Access to IT, printing and laminating facilities
Psychological support	Access to clinical psychologists for referral from allergy clinics Clinical psychologists trained in CBT and food allergy‐specific issues	Training of clinical psychologists in CBT and food allergy‐specific support	Number of food allergic patients in need of psychological support and their families seen by clinical psychologist (in relation to demand) Number of clinical psychologists with experience in seeing and supporting food allergic patients	Costs of training more clinical psychologists in general and on basics of allergic disease and integrating them in clinical services
Allergen‐specific immunotherapy	Facilities and staff trained in delivering AIT and in treatment of anaphylaxis Capacity of clinical services to accommodate multiple visits and ad‐hoc phone calls (especially for OIT and SLIT)	Training of HCP on performance of immunotherapy and treatment of anaphylaxis	Number of patients treated with allergen‐specific immunotherapy and proportion of referred patients	Costs of AIT and associated staff resources Training of HCP Space in clinical services with an appropriate setting for AIT
Biological treatments	Facilities and staff trained in treatment of anaphylaxis Capacity of clinical services to accommodate additional visits	Training of HCP on delivering biologic treatment and in educating patients for home‐based treatment and treatment of anaphylaxis	Number of patients treated with biologics and proportion of referred patients	Costs of biologics Training of HCP

Abbreviations: AIT, allergen‐specific immunotherapy; HCP, heathcare professional; CBT, cognitive behavioural therapy; IT, information technology; OIT, oral immunotherapy; SLIT, sublingual immunotherapy.

## 5 CONCLUSION

Once the diagnosis of IgE‐mediated food allergy has been confirmed, information about the culprit allergen and allergen avoidance measures, together with appropriate dietary advice allowing a balanced diet and continued consumption of safe foods that may have been avoided, are the first steps. Prescription of medications required to treat accidental allergic reactions and a written treatment plan together with education about recognition of allergic symptoms and administration of said medication, including intra‐muscular adrenaline using an auto‐injector are essential measures. In addition to these, psychological support may be required and immunomodulatory treatments, including allergen‐specific immunotherapy and biologics, may be indicated. For peanut allergy, oral immunotherapy is recommended and sublingual and epicutaneous immunotherapy are suggested; whereas for egg and milk allergies, only oral immunotherapy is suggested, generally after 4 years of age, when the chances of natural resolution are lower. Omalizumab is also suggested for the management of IgE‐mediated food allergy from 1 year of age, but this has only received regulatory approval in the USA. Food allergy management involves shared decision‐making by the physician and the patient and the family. Further research is highly needed to obtain evidence to support disease‐modifying therapeutic options in food allergy, using standardised study design and outputs, that are centred on patient experience and benefit.

## AUTHOR CONTRIBUTIONS

AFS, IS, CR and GdT wrote the manuscript based on the online meetings of the expert group. All authors critically reviewed the manuscript and approved its final version.

## FUNDING INFORMATION

European Academy of Allergy and Clinical Immunology.

## CONFLICT OF INTEREST STATEMENT

A.F. Santos reports grants from Medical Research Council (MR/M008517/1; MC/PC/18052; MR/T032081/1), Food Allergy Research and Education (FARE), the Immune Tolerance Network/National Institute of Allergy and Infectious Diseases (NIAID, NIH), Asthma UK (AUK‐BC‐2015‐01), BBSRC, Rosetrees Trust and the NIHR through the Biomedical Research Centre (BRC) award to Guy's and St Thomas' NHS Foundation Trust, during the conduct of the study; personal fees from Thermo Scientific, Nutricia, Infomed, Novartis, Allergy Therapeutics, Buhlmann, as well as research support from Buhlmann and Thermo Fisher Scientific through a collaboration agreement with King's College London. C. Riggioni reports academic grants for the study of immunotherapy in food allergic children from the Spanish Society of Paediatric Allergy (SEICAP) and the National University of Singapore. H. A. Brough reports research grants from NIH (NAIAD), Aimmune and DBV Technologies, and speaker honoraria fees from DBV Technologies, GSK and Sanofi outside of the submitted work. I. Agache reports Deputy Editor Allergy and Associate Editor Clinical and Translational Allergy. A. Fiocchi reports research grants from Ferrero, Hipp, Sanofi, Novartis, Astrazeneca and DBV, fees for presentations and advisory boards for Danone, Abbott, Ferrero, Stallergenes and Novartis. H. Fisher reports employment at Sanofi, outside of the submitted work. D. Fleischer reports research grants to institution from ARS Pharmaceuticals and DBV Technologies; unpaid advisory board member for Food Allergy & Anaphylaxis Connection Team and the National Peanut Board; royalties from UpToDate; consultation fees as a member of physician/medical advisory boards to Aquestive, ARS Pharmaceuticals, Bryn Pharma, DBV Technologies, Genentech, and Nasus; and speaker fees from Genentech, outside the submitted work. B. Ballmer‐Weber reports personal fees for presentations and advisory boards from Thermo Fisher Scientific, Novartis, ALK, Allergopharma, Menarini, Sanofi, MSD, Aiummune. S. Halken reports personal fees from ALK, personal fees from Mead Johnson, personal fees from Viatris, personal fees from GSK, outside the submitted work. S. Lau receives grants from Novartis, DBV, Infectopharm as deputy PI and a grant from the German Research Foundation (DFG). SL received personal fees from Allergopharma, ALK, Viatris, Lilly, DBV, GSK, Leo Pharma and Sanofi‐Aventis. P. Smith has received investigator initiated funding from GSK, Hyloris and Sanofi. C. G Mortz report research grant from Novartis and Thermo Fisher Scientific. B. Eberlein reports research support from Bühlmann. D. Fleisher reports Grant/research support: Aimmune Therapeutics, DBV Technolgies. Consultant: Aquestive Therapeutics, ARS Pharma, DBV Technologies, Nasus Pharma Genentech’ Royalties: UpToDate. M. Jutel reports: Personal fees form Allergopharma, ALK‐Abello, Stallergenes, Anergis, Allergy Therapeutics, Leti, HAL, GSK, Novartis, Teva, Takeda, Chiesi, Pfizer, Regeneron, Astra‐Zeneka, Lallemand, Shire, Celltrion Inc., Genentech, Roche, Verona, Lek Pharmaceuticals, Arcutis Biotherapeutics, FAES FARMA outside of submitted work. Deputy Editor in chief Allergy, Associate Editor CTA. C. Bindslev‐Jensen reports material for IgE analyzes from Thermofisher; Lecture fee from Alk‐Abello; advisory board fees from ALK and Novartis research grants from Ionis, Allakos, Novartis. E. F. Knol reports Research grants from Stichting Astma Bestrijding and European Union. Research support from Euroimmune, speakers fee from Thermo Fisher Scientific, Hycor, Sanofi and GSK. C. Jones reports Research grants from the National Institute for Health and Care Research, Food Standards Agency and honoraria from National Institute for Health and Care Research, Allergy UK and Danone/Nutricia. C. Gray reports speaker or advisory board fees from Nutricia, Thermofisher, Kenvue, Viatris and Sanofi. G. Du Toit reports grants and personal fees from Aimmune, grants and personal fees from DBV, personal fees from FARE, grants from NIH‐NIAID, grants and personal fees from Novartis, outside the submitted work. G. Roberts reports Research funding from National Institute of Health and Food Standards Agency. President of British Society of Allergy and Clinical Immunology.

H. Sampson reports funding to his institution for grants from NIH/NIAID and has received consulting fees from DBV Technologies, S. A., N‐Fold Therapeutics, LLC, and Siolta, Inc., and stock options from DBV Technologies and N‐Fold Therapeutics. S. Del Giacco has received speaker and consultancy fees from AstraZeneca, Chiesi, CSL‐Behring, GSK, Novartis, Sanofi, Stallergenes and unrestricted research grants from Novartis and GSK, all outside this work. G. N. Konstantinou is or recently was a speaker and/or advisor for and/or has received research funding from AstraZeneca, Chiesi, GSK, Menarini, Novartis, Nutricia, Pfizer, Sanofi, Takeda, TEVA and Vianex. C. Nilsson reports grants to institution from Aimmune Therapeutics a Nestlé Company, Lecture fees from ALK, Themofisher, GSK. A. Muraro declared the receipt of consultation or speakers' fees for Viatris, Aimmune, DVB Technologies, Nestlè Health Sciences, ALK, Stallergenes, Novartis, Sanofi. Regeneron. E. Untersmayr reports grants from Desentum Oy; and received personal speaker fees from Nutrica, AllergoPharma, MacroArray Diagnostics. B. P. Vickery reports grants from Abbott, grants and personal fees from Aimmune, grants from Alladapt, personal fees from AllerGenis, personal fees from Aravax, grants and personal fees from DBV, grants and personal fees from FARE, grants from Genentech, stock options from Moonlight Therapeutics, grants from NIH‐NIAID, grants and personal fees from Novartis, personal fees from Reacta Biosciences, grants and personal fees from Regeneron, personal fees from Sanofi, grants from Siolta, outside the submitted work. M. Worm declares the receipt of honoraria or consultation fees by the following companies: Novartis Pharma GmbH, Sanofi‐Aventis Deutschland GmbH, DBV Technologies S.A, Aimmune Therapeutics UK Limited, Regeneron Pharmaceuticals, Inc., Leo Pharma GmbH, Boehringer Ingelheim Pharma GmbH &Co.KG, ALK‐Abelló Arzneimittel GmbH, Lilly Deutschland GmbH, Kymab Limited, Amgen GmbH, Abbvie Deutschland GmbH & Co. KG, Pfizer Pharma GmbH, Mylan Germany GmbH (A Viatris Company), AstraZeneca GmbH, Lilly Deutschland GmbH and GlaxoSmithKline GmbH & Co. KG. B. Vlieg–Boerstra received research funding from Nutricia, consulting or speaker's fees from Marfo Food groups, Nestlé, Abbott, Nutricia and Vinimini. J. Schwarze reports personal consulting fees from Aimmune and Sanofi, congress sponsorship from ALK, research grants from NIHR and UKRI‐MRC. K. Hoffmann‐Sommergruber reports grant support from Government of Lower Austria (DARC) and consultancy fees from COMPARE Database. K. Beyer reports grants from the German Research Foundation, the Federal Ministry of Education and Research, the Federal Ministry of Food and Agriculture as well as from Aimmune, Danone/Nutricia, DBV, Hipp, Hycor, Infectopharm and Novartis; she received personal fees from Aimmune, Akademie Fresenius, Allergy Therapeutics, ALK, Danone/Nutricia, Hipp, Hycor, Infectopharm, Kantar Health, Limbach Gruppe, Mylan/Meda/Mice, Nestle, Novartis, Sonic Health Care and ThermoFisher. K. P. Perrett has received research grants from National Health & Medical Research Council of Australia, Immune Tolerance Network (NIH), Aravax, DBV Technologies, Novartis and Siolta and consultant fees from Aravax, paid to their institution, outside the submitted work. L. O'Mahony reports Consultancy with Precision BioticsAlimentary Health, grants from GlaxoSmithKline and Chiesi, and participation in speaker bureau for Nestle, Yakult, Reckitt and Abbott. L. Oliveira reports Speakers fee from Thermo Fisher Scientific, Nutricia, Sanofi and Takeda. Dunn‐Galvin declares the receipt of honoraria or consultation fees from Novartis, DBV, Aimmune, Nestle. M. Alvaro‐Lozano reports Honoraria or consultation fees from ALK‐Abello, FAES Pharma, LETI Pharma, Merck, Aimmune, DBV Technologies, Allergy Therapeutics, Stallergenes, Diater, Novartis, Uriach, Nestle and Sanofi Genzyme. Grants from SEICAP, SCAIC. M. Fernandez‐Rivas reports research support from Instituto de Salud Carlos III, Spanish Government, Aimmune Therapeutics, Diater and Novartis; Speaker and Advisory Board honoraria from Aimmune Therapeutics, DBV, Diater, Ediciones Mayo, EPG Health, GSK, HAL Allergy, MEDSCAPE; Novartis, Reacta Healthcare, SPRIM. M. Ebisawa reports Speaker and Advisory Board honoraria from Viatris, Novartis, Sanofi, and ARS‐Pharmaceuticals. M. Marshisotto reports advisory roles to IFPIES, National Peanut Board, Novartis Patient Advisory Board, GA2LEN ANACare and University of Michigan. N. Papadoupoulos reports Research Support from Capricare, Nestle, Numil, REG, Vianex. Speaker and Advisory Board honoraria from Abbott, Abbvie, Astra Zeneca, GSK, HAL, Medscape, Menarini/Faes Farma, Mylan, Novartis, Nutricia, OM Pharma, Regeneron/Sanofi. P. Smith reports Speaker and advisory board honoraria from Nestle Nutrition Institute. Investigator initiated research funding from GSK and Sanofi. Vitaris advisory board honoraria. P. Eigenmann reports Speaker and advisory board honoraria: DBV technologies, Novartis, ThermoFisher Scientific, Nestlé Health Sciences, Synlab, GSK; Stocks and Stock options: DBV technologies. R. Peters reports research grants from the National Health & Medical Research Council of Australia, and research support from ThermoFisher, paid to their institution, outside the submitted work. R. van Ree reports Consultancies for HAL Allergy, Citeq, Angany, Reacta Helathcare, Mission MightMe, AB Enzymes, The Protein Brewery; speaker's fees from HAL Allergy, ALK and Thermo Fisher Scientific; stock options from Angany. R. Meyer honoraria from academic lectures and consultancy fees from Nutricia/Danone, Abbott Laboratories, Nestle Clinical Nutrition, Reckitt Benckiser and Else Nutrition. T. Eiwegger reports to act/recently acted as local PI for company sponsored trials by DBV Therapeutics, Greer Stallergens, and sub‐investigator for Regeneronand ALK‐Abelló. He/his lab received unconditional/in‐kind contributions from Macro Array. Diagnostics and ALK‐Abelló and he is co‐I in an investigator‐initiated trial with in‐kind support from Novartis. He holds advisory board roles for ALK‐Abelló, and Nutricia/Danone. TE reports lecture fees from Novartis, ThermoFisher, Nutricia/Danone, MADX, ALK‐Abelló. T. Werfel has received institutional grants from LEO Pharma and Novartis, has performed consultancies for Abbvie, Almirall, Janssen, Galderma, LEO, Lilly, Novartis, Pfizer, Sanofi‐Regeneron and has lectured at events sponsored by Abbvie, Janssen, Celgene, Galderma, LEO Pharma, Lilly, Sanofi and Novartis. I. Skypala—honoraria from ThermoFisher, Royal College of General Practitioners and Touch Independent Medical Education. JOBH declares research funding from DBV Technologies, Johnson& Johnson, Consultancy with Camallergy, speaker fees from Nutricia. A. Alvarez‐Perea declares the receipt of honoraria or consultation fees from ALK‐Abelló, Organon, Immunotek, DBV‐technologies, GSK and PI and sub‐investigator for company sponsored trials by Novartis, Leti, CEU‐San Pablo, Aimmune. The other authors have nothing to disclose.

## Data Availability

Data sharing is not applicable to this article as no new data were created or analyzed in this study.
